# LED light spectra influence the stimulation of mycelial growth and anticancer activity in *Hericium erinaceus* mycelium

**DOI:** 10.3389/ffunb.2025.1684852

**Published:** 2025-11-26

**Authors:** Preuk Chutimanukul, Siripong Sukdee, Pawarisa Phetkaew, Ornprapa Thepsilvisut, Onmanee Prajuabjinda

**Affiliations:** 1 Department of Agricultural Technology, Faculty of Science and Technology, Thammasat University, Khlong Luang, Pathumthani, Thailand; 2 Department of Applied Thai Traditional Medicine, Faculty of Medicine, Thammasat University, Khlong Luang, Pathumthani, Thailand

**Keywords:** cytotoxic activity, fungal physiology, medicinal mushroom, optimal cultivation, photobiology

## Abstract

**Introduction:**

*Hericium erinaceus* is a medicinal mushroom known for producing diverse bioactive metabolites with therapeutic potential. However, cultivation strategies aimed at enhancing both fungal yield and metabolite bioactivity, particularly through light-mediated physiological modulation, remain insufficiently investigated. This study explored the influence of different LED light spectra on the growth performance and cytotoxic potential of *H. erinaceus* mycelia cultivated on a nutrient-rich red sorghum substrate.

**Methods:**

Mycelia were cultivated for 30 days under four LED light spectra-blue, red, green, and RGB, compared to a control treatment (which was kept in darkness). Growth parameters, including radial growth rate, colonization speed, fresh weight, biomass increase, and mycelial density, were recorded at harvest. Ethanol extracts prepared from the mycelia of each treatment were tested for cytotoxic activity against SW480 colorectal cancer cells, HepG2 liver cancer cells, and normal colon epithelial cells (CCD-841 CoN), and IC_50_ values were determined.

**Results and Discussion:**

Blue light produced the most pronounced enhancement in growth performance, yielding the highest mycelial density (0.344 g/cm^2^), fresh weight (6.75 g), and biomass increase (12.28%), along with the fastest radial expansion and substrate colonization. Extracts from blue light–treated mycelia showed the strongest cytotoxic effects against SW480 (IC_50_ = 133.71 μg/mL) and HepG2 cells (IC_50_ = 114.84 μg/mL), while exerting minimal effects on normal CCD-841 CoN cells. These findings suggest that targeted light spectra can modulate fungal physiology, likely via photoreceptor-mediated pathways, to enhance both agronomic performance and production of cytotoxic metabolites. This study provides a framework for optimizing *H. erinaceus* cultivation for functional food and therapeutic applications.

## 1 Introduction

The global food system is a highly interconnected network influenced by various factors, including agriculture, economics, politics, environmental conditions, transportation, storage, and consumer demand. Ensuring its sustainability requires long-term planning, structural reforms, and innovative approaches, especially in the face of challenges such as food insecurity and environmental degradation (Béné et al., 2019; Bell et al., 2022). Mushrooms present a valuable solution due to their nutritional, economic, and biotechnological significance. They not only serve as an essential food source but also have extensive applications in the pharmaceutical industry, offering bioactive compounds with anticancer, antioxidant, and immune-enhancing properties. Beyond their role in nutrition, medicinal mushrooms contribute to sustainable food production and healthcare advancements, making them a crucial resource in addressing global food security and health challenges (Khan et al., 2024).

The species *Hericium erinaceus* is commonly known as “Yamabushitake” or “lion’s mane” (Yang et al., 2021; Chutimanukul et al., 2023a). This species was selected as the primary focus of the present study due to its dual applications in both culinary practices and traditional medicine (Friedman, 2015; Chutimanukul et al., 2023a). Notably, *H. erinaceus* is recognized not only for its nutritional value, being rich in high-quality protein, dietary fiber, vitamins, and essential minerals, but also for its diverse bioactive compounds that contribute to various health benefits (Zou et al., 2001; Marimuthu et al., 2016). The fruiting body of *H. erinaceus* has been reported to contain numerous bioactive compounds, including triterpenes, phenolic compounds, hericenones, and erinacines (Zou et al., 2001; Chutimanukul et al., 2023a). In recent years, this species has increasingly gained attention from the scientific community owing to its promising pharmacological properties, particularly its antioxidant potential and anticancer effects against gastrointestinal malignancies, among other therapeutic prospects (Wong et al., 2013; Phan et al., 2014). The cultivation of *H. erinaceus* is a resource-intensive and time-consuming process, typically spanning around two months. It involves three main stages: mycelium colonization, primordial formation, and fruiting body (Liang et al., 2013; Cheng et al., 2021). Traditionally, hardwood sawdust has been the primary substrate due to its moisture retention and carbon source (Tuomela et al., 2000). However, its slow decomposition and delayed nutrient release can impede early mycelial establishment, a crucial factor for successful mushroom cultivation (Chiang et al., 2017). As a result, researchers have explored alternative methods to enhance efficiency, necessitating structural modifications and innovative solutions (Chutimanukul et al., 2025). A promising approach that has garnered significant attention in mushroom cultivation is the use of grain-based substrates to accelerate the growth process. This method, inspired by traditional fermented foods such as “Japanese natto” and “Red rice yeast”, has facilitated the development of mushroom-fermented grain products (Liang et al., 2013; Cheng et al., 2021). Among various grain options, red sorghum has been identified as a particularly effective substrate, offering a synergistic combination of the bioactive properties of *H. erinaceus* and the inherent nutritional benefits of the grain (Liang et al., 2009a, 2009; Chiang et al., 2017). Red sorghum is a nutrient-dense and gluten-free grain, making it valuable in human nutrition for managing diabetes, as well as being widely used in functional foods (Devi et al., 2014). In mushroom cultivation, red sorghum is widely recognized as an excellent spawn substrate due to its small grain size, which allows a high number of inoculation points, and its capacity to support rapid and vigorous mycelial colonization (Kumbhar, 2012). Moreover, its high carbohydrate and protein content provides a rich source of energy and nutrients, promoting robust mycelial growth and enhancing the production of bioactive secondary metabolites in cultivated mushrooms (Narh Mensah et al., 2011).

In addition to substrate selection, light is a critical environmental factor influencing the growth and development of mushrooms (Chiang et al., 2017). It plays a fundamental role in regulating physiological processes, including mycelial expansion, fruiting body formation, and metabolic activity. Variations in light wavelength, intensity, and exposure duration can significantly impact yield, morphology, and the synthesis of bioactive compounds. These effects are mediated through light-sensing pathways that activate key developmental genes, ultimately shaping the growth and functional properties of the mushroom (Chao et al., 2019; Feng et al., 2023). For example, Chiang et al. (2017) demonstrated that distinct LED wavelengths differentially influenced the growth and metabolite profiles of *Cordyceps militaris* cultivated on brown rice: red light enhanced biomass and mannitol production, green light promoted cordycepin accumulation, and blue light favored adenosine synthesis, with red-blue combinations yielding the greatest overall productivity. Similarly, Park and Jang (2020) reported that blue light significantly altered fruit body morphology and proteomic expression in *Lentinula edodes*, increasing pileus size and thickness, modifying stipe development, and up-regulating proteins associated with energy metabolism and cell wall formation while down-regulating others. Collectively, these findings underscore light as a pivotal determinant of mushroom physiology and metabolite production. However, despite the advantages of LED lighting, its application in the cultivation of *H. erinaceus* remains unexplored, with limited research on its combined effects with grain-based substrates. This study investigates the impact of different LED light spectra and combined lighting treatments on the mycelial growth and anticancer activity against gastrointestinal cancer cells of *H. erinaceus* cultivated on red sorghum. The findings from this research aim to support the development of *H. erinaceus*-based functional foods as a future dietary supplement.

## 2 Materials and methods

### 2.1 Mushroom strain and substrate preparation

The *H. erinaceus* strain used in this study was isolated from fresh fruiting bodies cultivated at Thammasat University (Pathum Thani, Thailand). Tissue from the inner portion of the fruiting body was excised under sterile conditions and transferred to potato dextrose agar (PDA) plates. The plates were incubated at 25 ± 1 °C until the mycelium fully colonized the agar surface. Once the mycelium had fully colonized the PDA medium, actively growing mycelium from the edges of the colony was cut into small pieces and used to inoculate the red sorghum grain substrate.

Red sorghum grains were used as the primary cultivation substrate for *H. erinaceus* mycelium. The red sorghum is washed and then boiled for 15 minutes. After boiling, 55 grams of red sorghum are transferred into glass bottles with a diameter of 5 cm and a height of 10 cm. The bottles are then sterilized using an autoclave at 121 °C for 15 minutes to eliminate contaminants. After cooling to room temperature, each glass bottle was inoculated at the bottom corner with a 1×1 cm PDA block containing actively growing *H. erinaceus* mycelium, in order to monitor the mycelial growth from one corner to the opposite side of the bottle. The inoculated red sorghum bottles were incubated at 25 ± 1 °C under dark conditions for an initial incubation period of 7 days to allow the mycelium to establish and colonize the grains (**Supplementary Figure S1**).

### 2.2 Investigation of *H. erinaceus* mycelial development under different light spectra

#### 2.2.1 Experimental design and light treatments

This experiment was designed to investigate the effect of different light spectra on the mycelial growth of *H. erinaceus* cultured on red sorghum grains. The experiment consisted of four light treatments - blue light (wavelength 450 nm), green light (wavelength 520 nm), red light (wavelength 660 nm), and a combination of red, green, and blue light (RGB) (**Supplementary Figure S2**) - along with a control treatment where the cultures were maintained in complete darkness to verify the experimental results obtained from the use of LED light. The light intensity for all treatments was maintained at 40 ± 5 µmol m^-^²s^-^¹, with a daily exposure period of 8 hours (Chiang et al., 2017). Following this, the inoculated substrates were incubated under the designated light conditions at a temperature of 25 ± 1 °C (**Supplementary Figure S1**). The light sources used were LED panels, each calibrated to ensure uniform light distribution across the incubation area. The spectral intensity of each light source was verified using a quantum light meter (UPRTEK PG200N) to ensure consistency.

#### 2.2.2 Study of *H. erinaceus* mycelial growth rate

The mycelial growth rate on red sorghum was measured every 3 days after the initiation of light exposure. Measurements were conducted using a sterilized vernier caliper, recording the distance of mycelial extension from the inoculation point until the mycelium fully colonized the 5 cm length, extensively extending from the inoculation point and permeating through the substrate until complete colonization was achieved, corresponding to the size of the glass bottle. All measurements were taken at the same time of day to avoid variations caused by potential circadian effects.

#### 2.2.3 Study of mycelial fresh weight and %increase in mycelial growth

The biomass assessment of *H. erinaceus* mycelium cultivated on red sorghum was adapted from the method of Wang et al. (2024), focused solely on recording the fresh weight of the mycelium grown on the red sorghum. The weight measurement was performed after 30 days of cultivation. Fresh mycelial weight is estimated by a mass-balance approach in solid-state culture: the substrate is weighed before inoculation (FW_1_) and again after incubation (FW_2_); the approximate fresh mycelial biomass equals (FW_2_ − FW_1_).

The change in mycelial biomass was evaluated through the difference in fresh weight of the red sorghum substrate measured before and after incubation with *H. erinaceus*. Nevertheless, it primarily reflects physical biomass accrual and colonization, not instantaneous physiological activity. Initially, 55 g of red sorghum was weighed prior to inoculation to obtain the initial fresh weight (FW_1_). After the incubation period, the combined weight of the red sorghum and mycelium was recorded as the final fresh weight (FW_2_). The percentage increase in mycelial growth was calculated based on the difference between the final and initial weights, expressed relative to the initial weight, and converted to a percentage, according to the modified method of Irbe et al. (2022). This calculation was performed according to Equation:


$$\text{\% Mycelial Growth Increase}=\frac{\text{F}{\text{W}}_{2}-\text{F}{\text{W}}_{1}}{\text{F}{\text{W}}_{1}}\times 100$$

FW_1_ = Weight of red sorghum before it was incubated with the fungus.

FW_2_ = Weight of red sorghum and mycelium after incubation.

#### 2.2.4 Study of mycelial density

The determination of mycelial density in *H. erinaceus* serves as a quantitative indicator reflecting its growth capacity and can be divided into the following two steps:

##### 2.2.4.1 Determining the mycelial area

**Figure 1:**
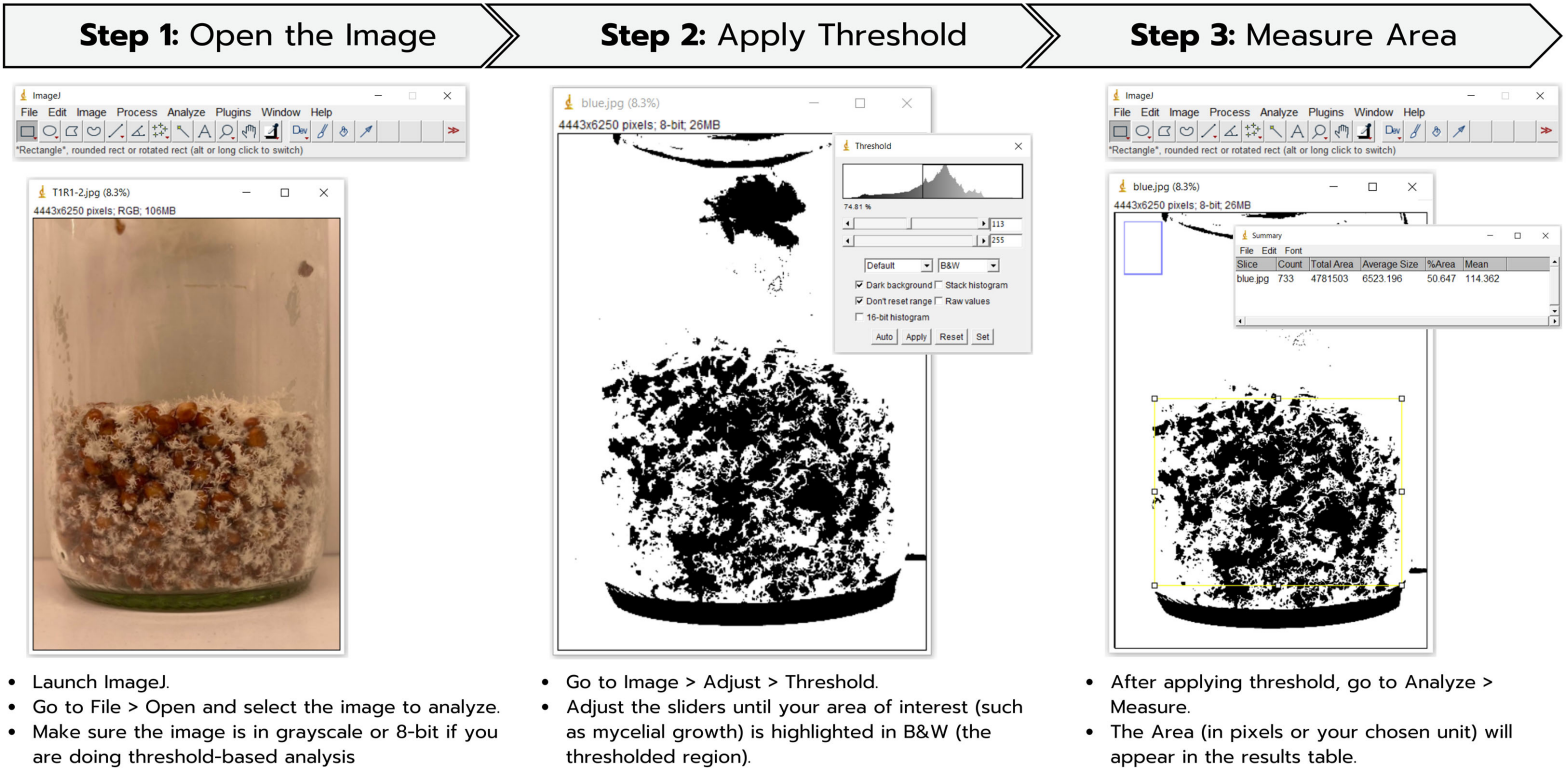
Determination of mycelial density by measuring the mycelial area of *H. erinaceus* using ImageJ.

Photographs of *H. erinaceus* mycelium in glass bottles were taken by rotating each bottle completely, and the obtained values were averaged. The images obtained were then processed using the ImageJ software (version 1.53t, National Institutes of Health, USA) to calculate the total mycelial area (Fukasawa and Kaga, 2020), with all analyses conducted under standardized imaging and processing conditions to ensure reproducibility. In the first step, images from each experiment are imported into the software. Then, the ImageJ software is used to calculate the percentage of white pixels, which represents the area covered by mycelial growth from photographs taken around all sides of the cultivation bottle, as shown in **Figure 1**. The resulting values are presented as %Area, calculated by averaging the measurements taken from all sides of the bottle.

The %Area derived from the ImageJ software is then utilized to determine the percentage of mycelial coverage in relation to the surface area of the culture bottle, following the equation:


$$\text{Mycelial area }=\frac{\;\%\text{Area}}{100}\times \;\text{Area of bottle }(\text{cm}²)$$

For each biological replicate, three independent images were taken at 120° intervals around the bottle, and the mean coverage area was calculated; the resulting measurement corresponds to the surface area of the circular base of the bottle. Therefore, the area was calculated using the formula A = πr². The cultivation bottles used for *H. erinaceus* mycelial growth had a diameter of 5 cm, corresponding to a radius (r) of 2.5 cm, resulting in a total surface area of approximately 19.63 cm².

##### 2.2.4.2 Mycelial density determination

In this method, mycelial density was determined according to the Equation below, where the fresh mycelial weight was obtained by subtracting the initial substrate weight (55 g) from the final weight after 30 days of cultivation. The mycelial growth area (cm²) was then measured, and the mycelial density (g/cm²) was calculated by dividing the fresh mycelial weight by the measured area. This approach, adapted from Agba et al. (2021), allows for the standardization of biomass per unit area, facilitating direct comparisons of *H. erinaceus* growth performance under varying light conditions.


$$\text{Mycelial density }=\frac{\text{ Fresh weight of mycelium }(\text{g})}{\text{Mycelial area }({\text{cm}}^{2})}$$

#### 2.2.5 Data analysis

The growth of *H. erinaceus* mycelium was subsequently used to create a heatmap, correlation plot, and principal component analysis (PCA) visualizing the development of mycelial colonization across different light conditions. This heatmap was created using Python 3.11.0, specifically with Matplotlib version 3.7.0.

### 2.3 *In vitro* assay for cytotoxic activity

#### 2.3.1 Preparation of ethanol extract

The *H. erinaceus* mycelium samples obtained from the light treatment experiments, which had fully colonized the red sorghum grains and for which growth data had been collected, together with the red sorghum grains used as the substrate, will be oven-dried at 60°C for 48 hours, ground into a fine powder, and stored in sealed containers until analysis. For extraction, 5 g of the dried powder was mixed with 25 mL of absolute ethanol (w/v) at room temperature. The mixture was filtered through Whatman^®^ Grade 1 qualitative filter paper, and the maceration process was repeated every three days over a total period of nine days. After the final extraction, the combined filtrates were concentrated by solvent evaporation under vacuum using a rotary evaporator (Rotavapor^®^ R-300, BUCHI, Flawil, Switzerland). The crude ethanol extract obtained was stored at 4°C until further use (Chutimanukul et al., 2023b).

#### 2.3.2 Human cell lines

The cell lines present in this study were obtained from ATCC (American Type Culture Collection) and consisted of three cell lines, including the human colorectal cancer cell line (SW480; CCL-228), which was cultured in Roswell Park Memorial Institute (RPMI) medium supplemented with 10% heat-inactivated Fetal Bovine Serum (FBS), 1% Penicillin-Streptomycin, and 2 g/L Sodium bicarbonate (NaHCO_3_); the hepatocellular carcinoma cell line (HepG2; HB-8045), which was cultured in Minimum Essential Medium (MEM) containing 10% heat-inactivated FBS, 1% Penicillin-Streptomycin, 10 mM HEPES, and 2.2 g/L NaHCO_3_; and the normal human colon epithelial cell line (CCD 841 CoN; CRL-1790), which was cultured in Eagle’s Minimum Essential Medium (EMEM) supplemented with 10% heat-inactivated FBS, 1% Penicillin-Streptomycin, and 1.5 g/L NaHCO_3_. To maintain exponential cell growth during experiments and to ensure a linear correlation between absorbance at 492 nm and cell number in the SRB assay, optimal seeding densities were established for each cell line based on their respective growth characteristics.

#### 2.3.3 Sulforhodamine B assay

**Figure 2:**
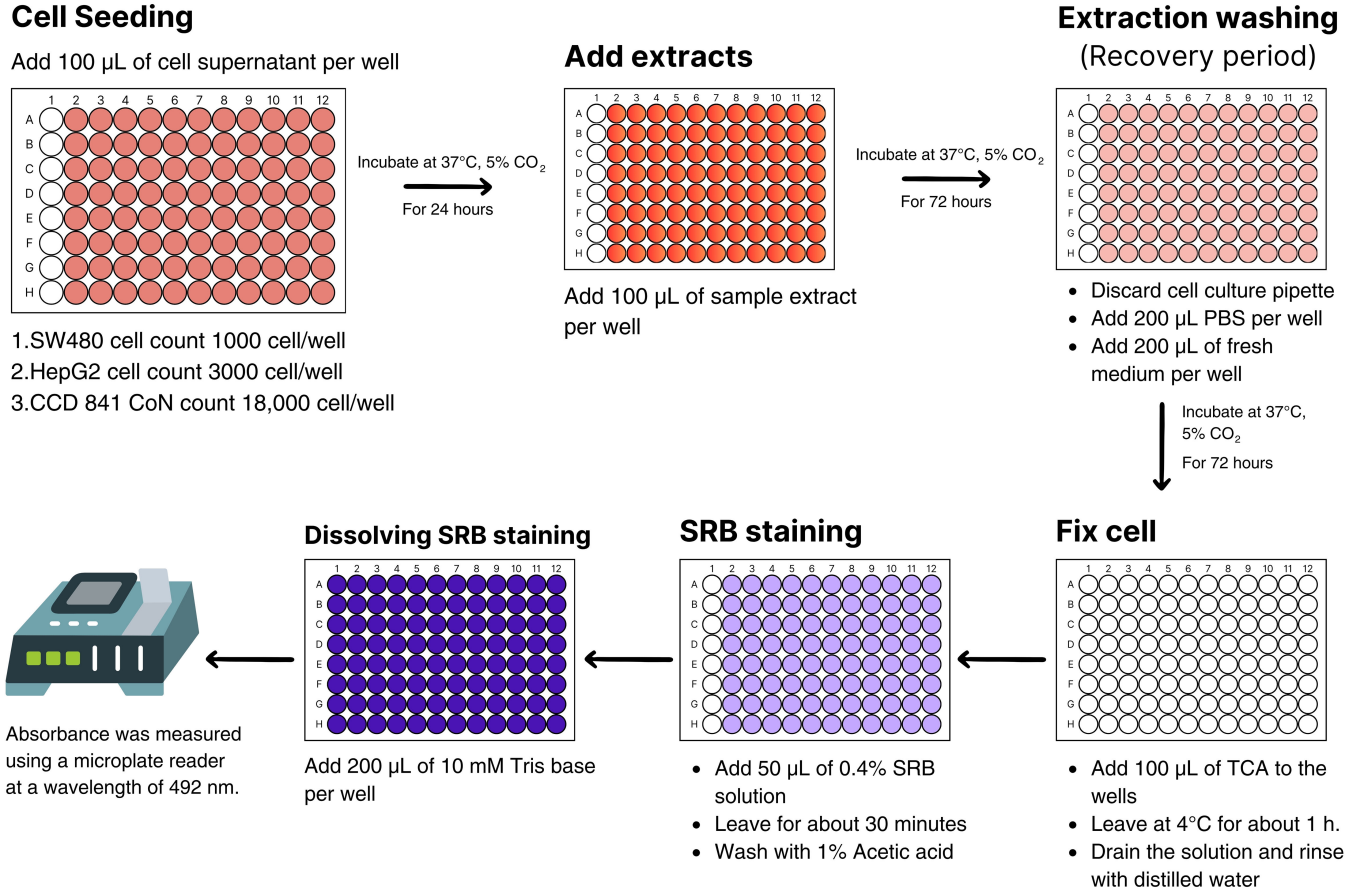
*In vitro* evaluation of cytotoxic activity against cancer cells using the Sulforhodamine B assay.

The antiproliferative SRB assay was performed to assess growth inhibition by a colorimetric assay that estimates cell number indirectly by staining total cellular protein with the dye SRB (Suthisamphat et al., 2020). Extracts of *H. erinaceus* mycelium obtained under all light treatments: blue, green, RGB, and red, as well as the untreated control, together with red sorghum grains as an additional control for the cell-based assays, were evaluated for cytotoxic activity against SW480, HepG2, and CCD 841 CoN cell lines. The cells were cultured in appropriate culture media in cell culture flasks, allowing them to adhere to the flask surface. Cytotoxicity testing was conducted according to the procedure illustrated in **Figure 2**, which was created using Canva Pro. Cells were seeded into 96-well microplates at a density of 1,000 cells/well for SW480, 3,000 cells/well for HepG2, and 18,000 cells/well for CCD 841 CoN, with a volume of 100 µL per well. The cells were incubated and maintained at 37°C with 5% CO_2_ in a CO_2_ incubator to allow attachment and monolayer formation in 24 hours. Subsequently, ethanol extracts were added to the culture media at various concentrations: 300, 200, 100, 50, 25, and 12.5 µg/mL, respectively, with 100 µL per well. Each concentration was tested in four replicate wells. The plates were then incubated in a CO_2_ incubator for 72 hours. After incubation, the culture media were gently removed, and the monolayer cells were washed once with PBS sterile. Then, 200 µL of fresh culture media was added, and the plates were incubated again for 72 hours to assess cancer cell survival, using non-treated and solvent controls as references. Cytotoxicity was determined using the Sulforhodamine B (SRB) assay. Viable cells were fixed with 40% Trichloroacetic acid (TCA), and stained with 0.4% SRB dye, and then the bound dye was solubilized with 10 mM Tris base. Absorbance was measured at 492 nm using a microplate reader. The absorbance data were used to perform statistical analysis and calculate the IC_50_ values from dose-response curves as an indicator of cytotoxic activity, using the Prism software. The experiments were conducted in triplicate to confirm the results (Itharat et al., 2004).

### 2.4 Statistical analysis

The experiment followed a completely randomized design (CRD), with each light treatment replicated 5 times (*n* = 5), each replication consisting of 3 bottles. Data were subjected to analysis of variance (ANOVA) with Duncan’s multiple-range test to determine the statistical significance of the effects of the light spectrum on mycelial growth.

## 3 Results

### 3.1 Comparative study of *H. erinaceus* mycelial growth rate under different light spectra

**Figure 3:**
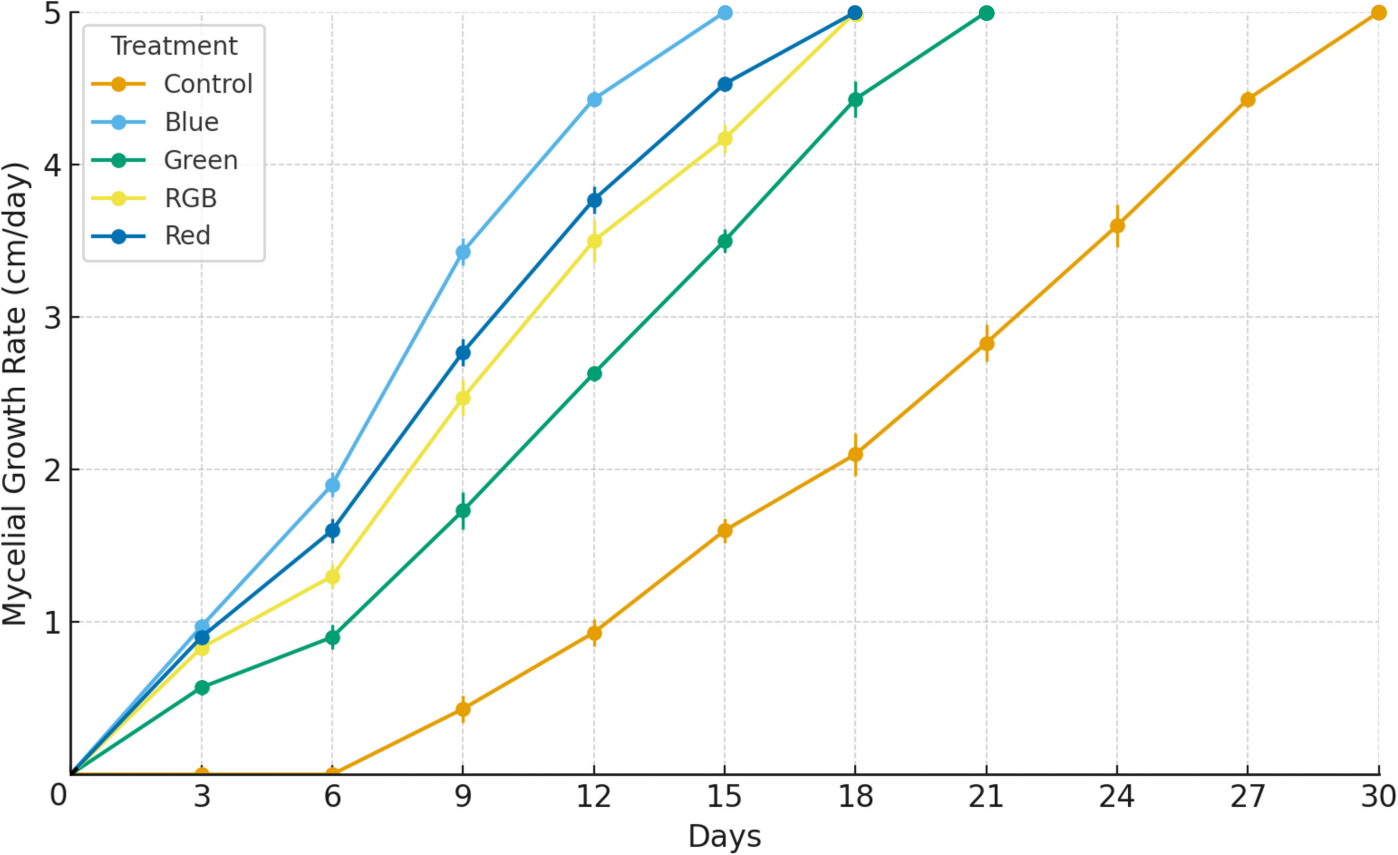
Effects of LED light treatments on the mycelial growth rate of *H. erinaceus* during a 30-day cultivation period. The maximum mycelial growth rate was standardized at 5 cm. Data are expressed as mean ± standard deviation (*n* = 5).

The growth of *H. erinaceus* mycelium under different light conditions was assessed by the time required for the mycelium to completely cover the culture surface. Cultures were maintained for 30 days, with measurements recorded every 3 days, and the maximum colony diameter was standardized at 5 cm. The results showed that mycelial growth progressively increased in all treatments during the early phase of incubation, followed by a plateau once the maximum growth diameter was reached. Among the treatments, blue light promoted the fastest mycelial expansion, reaching the maximum diameter of 5 cm by day 15, with a steep increase observed between days 6 and 12. The red and RGB treatments also exhibited relatively rapid growth, attaining 5 cm on day 18. Conversely, the control treatments demonstrated noticeably slower mycelial development, achieving the maximum diameter only by day 30, with comparatively lower average growth rates across the cultivation period (**Figure 3**; **Supplementary Table S1**). Throughout the experiment, the blue light treatment consistently recorded the highest mean mycelial growth rate and the lowest variation, followed by red and RGB, while the control treatments displayed delayed and slower progression. These findings confirm that the growth performance of *H. erinaceus* mycelium is markedly influenced by light spectrum, with distinct differences in growth rate and time to maximum colony diameter among treatments.

### 3.2 Effect of different light spectra on mycelial fresh weight and %increase in mycelial growth

**Figure 4:**
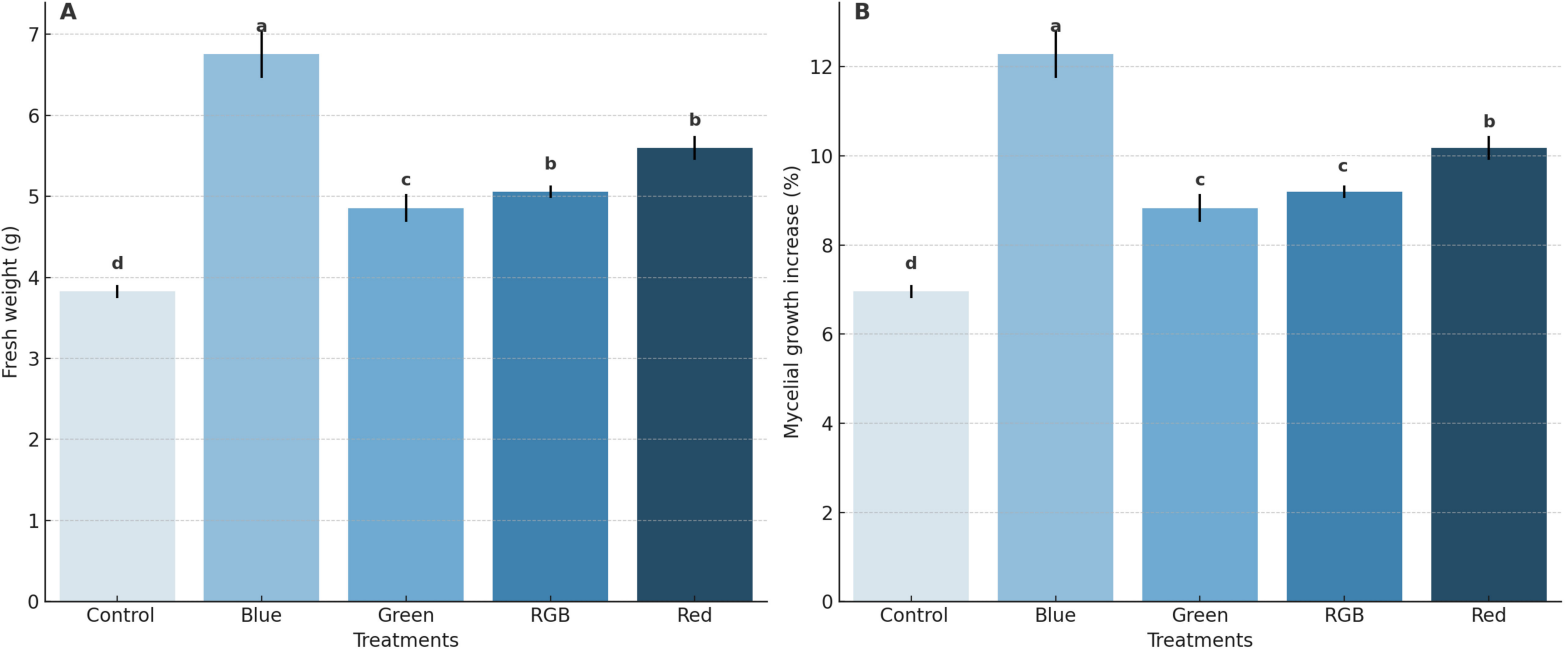
Effects of LED light treatments on the growth performance of *H*. *erinaceus* on **(A)** Fresh weight of mycelial biomass and **(B)** Percentage increase in mycelial growth. Bars represent means ± standard deviation (*n* = 5). Different letters above the bars indicate statistically significant differences among treatments based on DMRT (*p* < 0.05).

The effects of different light treatments on both the fresh weight of *H. erinaceus* mycelium and the %increase in mycelial growth. The results indicate that blue light treatment resulted in the highest fresh weight of *H. erinaceus* mycelium, with an average fresh weight of 6.75 g. This was significantly higher than the control treatment, which exhibited the lowest average fresh weight at 3.83 g. The green, RGB, and red-light treatments exhibited moderate fresh weights of *H. erinaceus* mycelium at 4.86 g, 5.06 g, and 5.60 g, respectively (**Figure 4A**; **Supplementary Table S1**). In terms of %mycelial growth increase, the blue light treatment also demonstrated the highest increase, with an average of 12.28%. Conversely, the control treatment displayed the lowest growth increase, averaging only 6.96%. The green, RGB, and red-light treatments showed moderate growth increases of 8.83%, 9.19%, and 10.18%, respectively (**Figure 4B**; **Supplementary Table S1**). Overall, these findings demonstrate that blue light treatment most effectively promotes both the fresh weight and the percentage increase of *H. erinaceus* mycelial growth compared to other light treatments.

### 3.3 Comparative study of mycelial density under different light spectra

**Figure 5:**
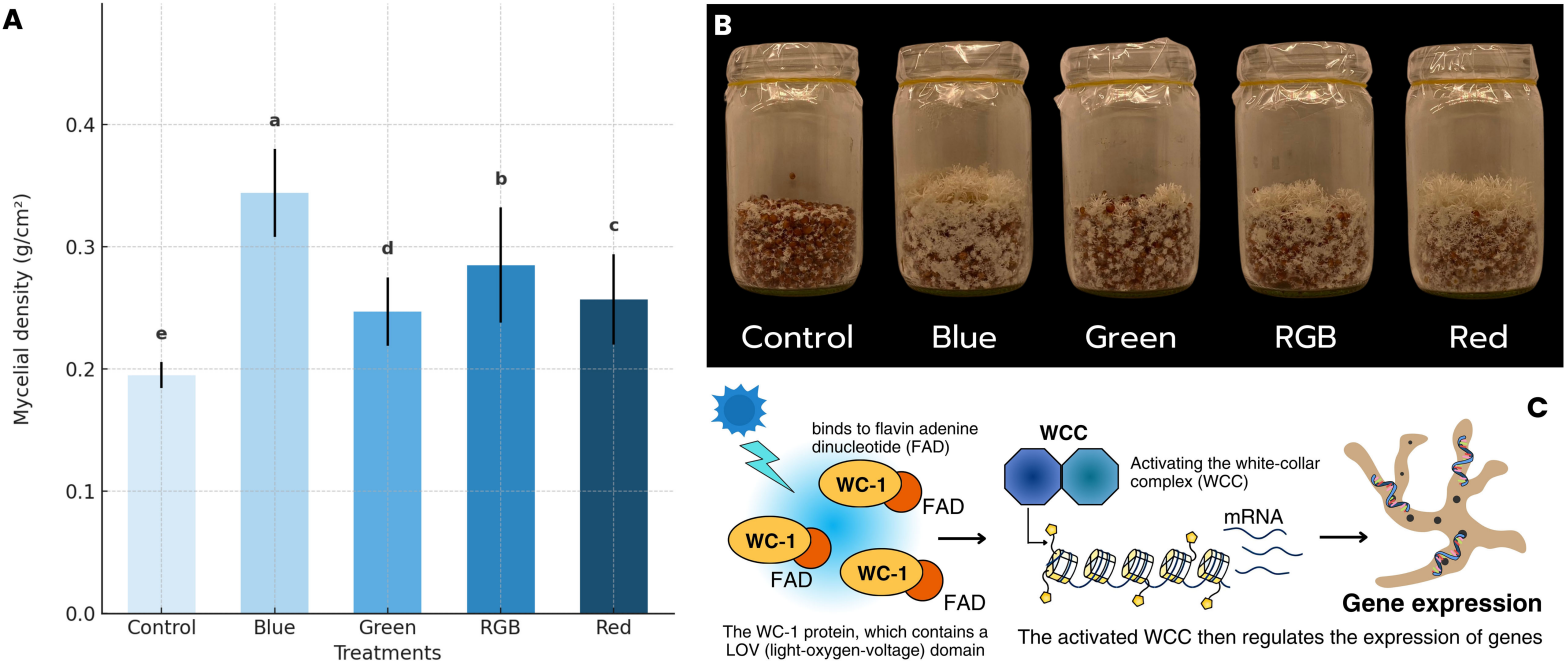
The effect of light spectrum on **(A)** Mycelial density, **(B)** Mycelial growth characteristics, and **(C)** Blue light receptor response mechanism under light treatments.

The average *H. erinaceus* mycelium density across different light treatments was analyzed, and the results are presented in **Figure 5A**; **Supplementary Table S1**. Among the treatments, the blue light condition exhibited the highest average *H. erinaceu*s mycelium density, with a value of 0.344 g/cm². This result supports previous findings indicating that blue light plays a critical role in fungal development and biomass accumulation. In contrast, the control treatment, which was cultured under dark conditions, showed the lowest average density at 0.195 g/cm². This suggests that the absence of controlled light spectra may limit mycelial growth. The green, RGB, and red-light treatments resulted in intermediate levels of *H. erinaceus* mycelium density, with average values of 0.247 g/cm², 0.257 g/cm², and 0.285 g/cm², respectively. These findings are consistent with studies indicating that fungal species respond differently to red and green light depending on their photoreceptor composition and ecological adaptations. In addition, the characteristics of the *H. erinaceus* mycelium, as shown in **Figure 5B**, the morphological characteristics of *H. erinaceus* mycelium clearly demonstrate the influence of different light treatments, control, blue, green, RGB, and red on the development of secondary mycelia. Consistent with the quantitative analysis of mycelial density, the blue light treatment produced the most extensive and compact mycelial network, characterized by thick, fluffy, and highly branched hyphae that covered nearly the entire substrate surface. This observation aligns well with the measured data, indicating that blue light yielded the highest mycelial density among all treatments. The RGB condition also promoted vigorous colonization and branching, though slightly less dense than that observed under blue light. In contrast, the control group maintained in darkness exhibited sparse hyphal coverage and thinner filamentous structures, corresponding to the lowest recorded density. Meanwhile, the green and red light treatments showed moderate mycelial expansion and aggregation, consistent with their intermediate biomass values. Notably, slight primordia-like formations were occasionally observed under the green and RGB treatments, suggesting early signs of differentiation toward fruiting initiation. Collectively, these findings indicate that blue light most effectively stimulates secondary mycelial formation and structural development of *H. erinaceus*, underscoring its potential role in optimizing illumination conditions for enhanced mycelial growth prior to the fruiting phase. Additional observations regarding molecular responses are summarized in **Figure 5C** (created using Canva Pro), which depicts the detected light-responsive behavior of the WC-1 protein containing an LOV (light-oxygen-voltage) domain bound to flavin adenine dinucleotide. The figure shows the molecular components identified in the light response pathway under blue-light treatment.

### 3.4 Multivariate analysis of mycelial growth parameters under different light treatments

Multivariate statistical approaches were applied to evaluate how different light spectra (Control, Blue, Green, Red, and RGB) influenced multiple growth parameters of *H. erinaceus* mycelium simultaneously. By integrating correlation analysis, hierarchical clustering, and PCA biplot, the analysis captured interrelationships among mycelial growth rate, mycelial weight, %mycelial growth increase, and mycelial density.

**Figure 6:**
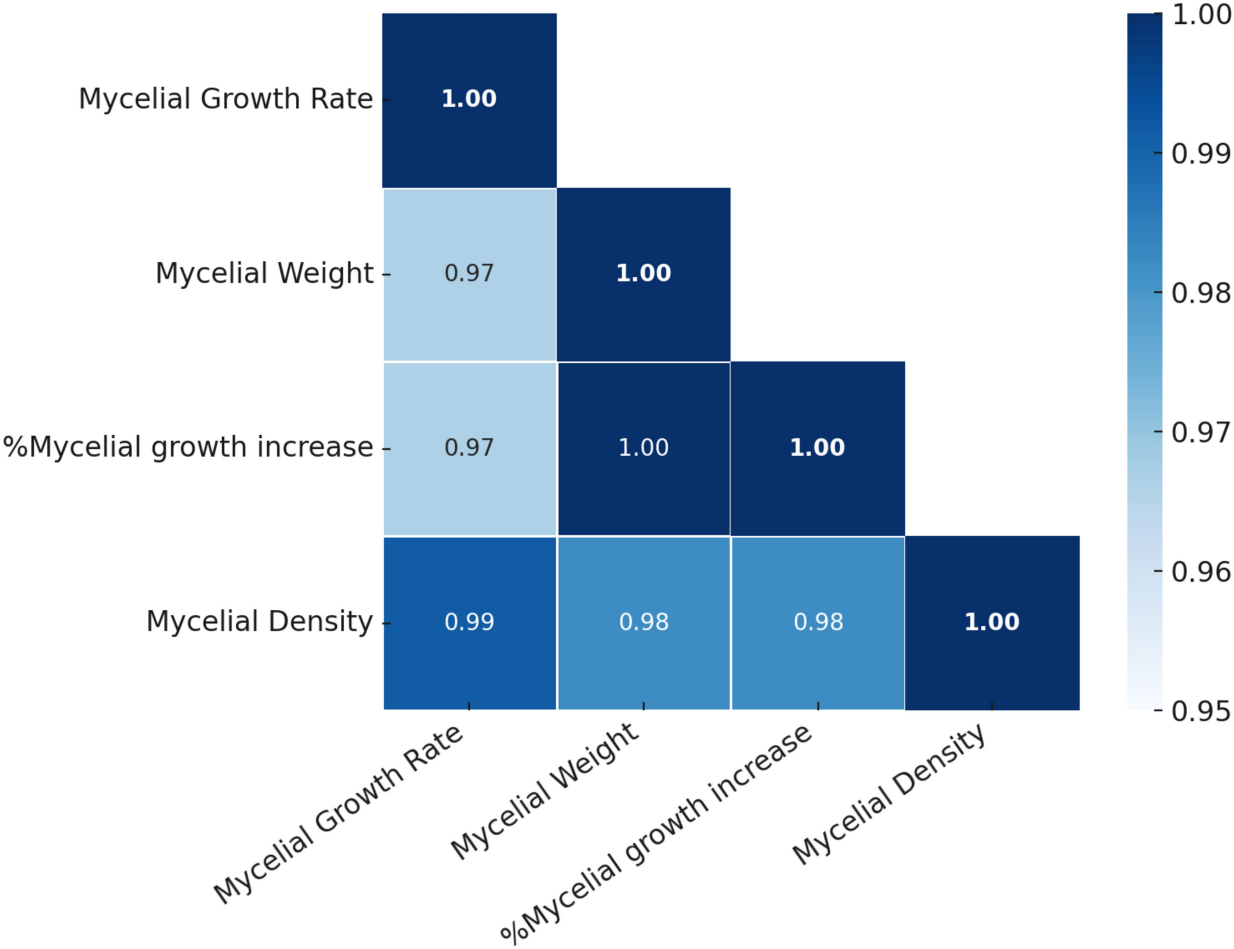
Correlation matrix of mycelial growth parameters of *H. erinaceus* under different LED light treatments. The heatmap displays Pearson correlation coefficients among mycelial growth rate, mycelial weight, percentage mycelial growth increase, and mycelial density. Color intensity represents the strength of correlation, with darker shades indicating stronger positive associations.

The correlation plot illustrates the relationships between key mycelial growth parameters, including mycelial growth rate, mycelial weight, %mycelial growth increase, and mycelial density, under different light exposure conditions. The analysis reveals a consistently strong positive correlation among all variables (*r* > 0.95), indicating that these parameters are closely interrelated. The strongest correlation (*r* = 1.00) is observed between mycelial weight and %mycelial growth increase, suggesting that the relative increase in mycelial growth directly translates into increased fresh weight. This reflects the fundamental biological connection between growth, expansion, and biomass accumulation. Furthermore, mycelial growth rate and mycelial density also show a very high correlation (*r* = 0.99), implying that faster radial growth contributes significantly to the accumulation of denser mycelial networks. This positive link highlights the structural development of the mycelial mat, where enhanced outward growth corresponds with increased mycelial compaction and biomass per unit area. Additionally, the correlations between mycelial growth rate and both %mycelial growth increase and mycelial weight (*r* = 0.97) further confirm that radial growth rate is tightly linked to both biomass production and the relative increase in mycelial growth over time (**Figure 6**). These findings suggest that growth rate is not only a primary driver of expansion but also a critical determinant of overall biomass accumulation and quality.

**Figure 7:**
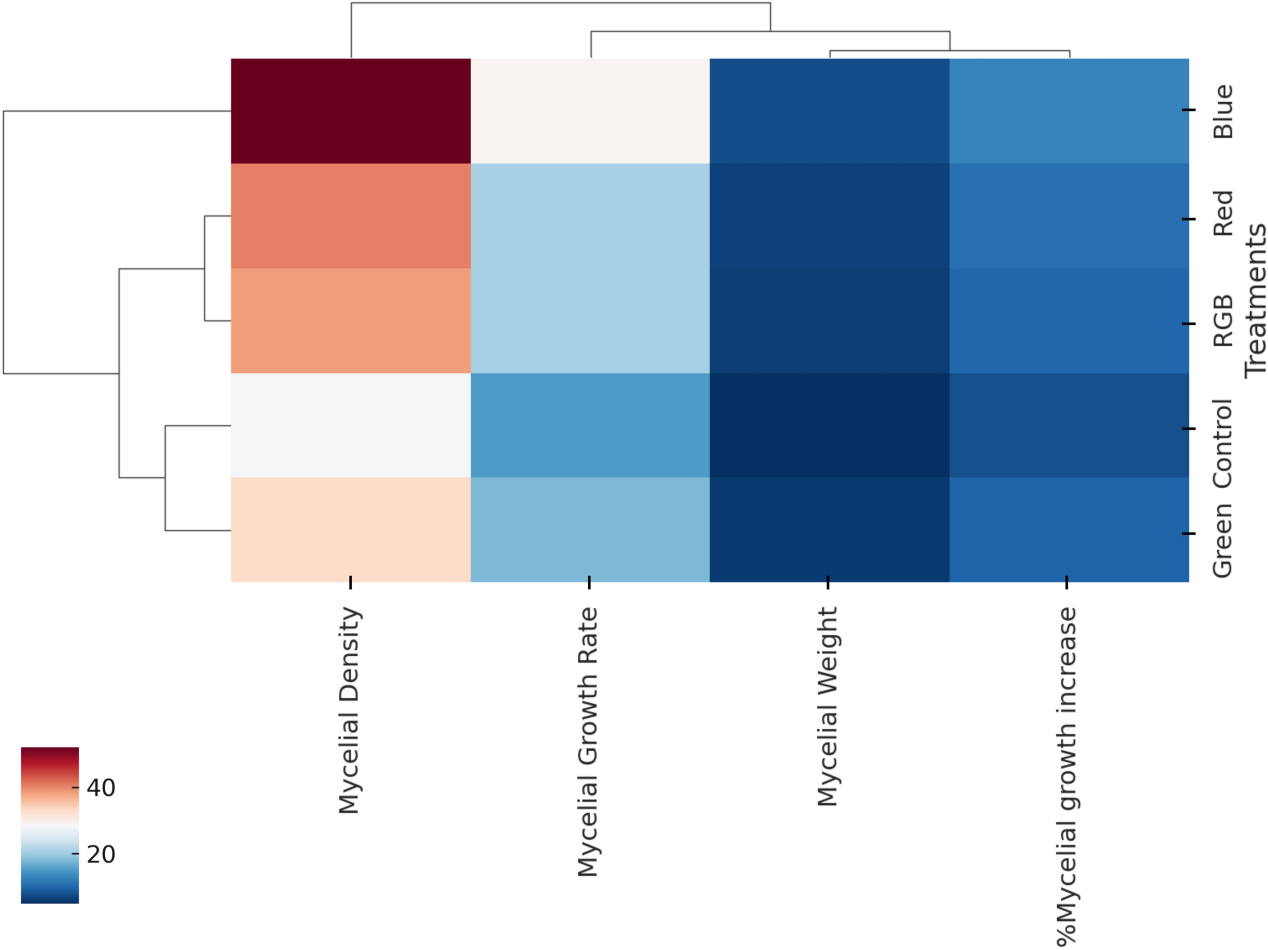
Hierarchical clustering heatmap of mycelial growth parameters of *H. erinaceus* under different LED light treatments. The heatmap depicts the relationships between treatments (control, blue, green, RGB, and red) and growth parameters (mycelial density, mycelial growth rate, mycelial weight, and percentage mycelial growth increase).

Hierarchical clustering combined with a heatmap (**Figure 7**) was employed to visualize variations in *H. erinaceus* mycelium growth parameters across the five experimental treatments (control, blue, red, green, and RGB), enabling the grouping of treatments with similar response profiles while simultaneously clustering the measured parameters according to their correlation patterns. The analysis revealed that the blue light treatment was distinctly separated from the other treatments, exhibiting the highest values for *H. erinaceus* mycelial growth rate, mycelial weight, %mycelial growth increase, and mycelial density, whereas the red light and RGB treatments clustered more closely, showing moderate increases in growth-related parameters compared with the control, and the Control and green light treatments were grouped together, reflecting relatively lower values across most parameters, particularly in mycelial density and %growth increase. Among the parameters, *H. erinaceus* mycelial growth rate and mycelial density were strongly correlated, forming a closely linked sub-cluster, while mycelial weight and %mycelial growth increase grouped separately, suggesting partially independent variation from growth rate and density, thereby indicating that different light spectra influenced distinct aspects of *H. erinaceus* fungal biomass accumulation and hyphal development. Overall, the heatmap and dendrogram highlight that blue light provided the most pronounced stimulation of *H. erinaceus* mycelial growth, while green light and the control condition yielded the least favorable growth outcomes.

**Figure 8:**
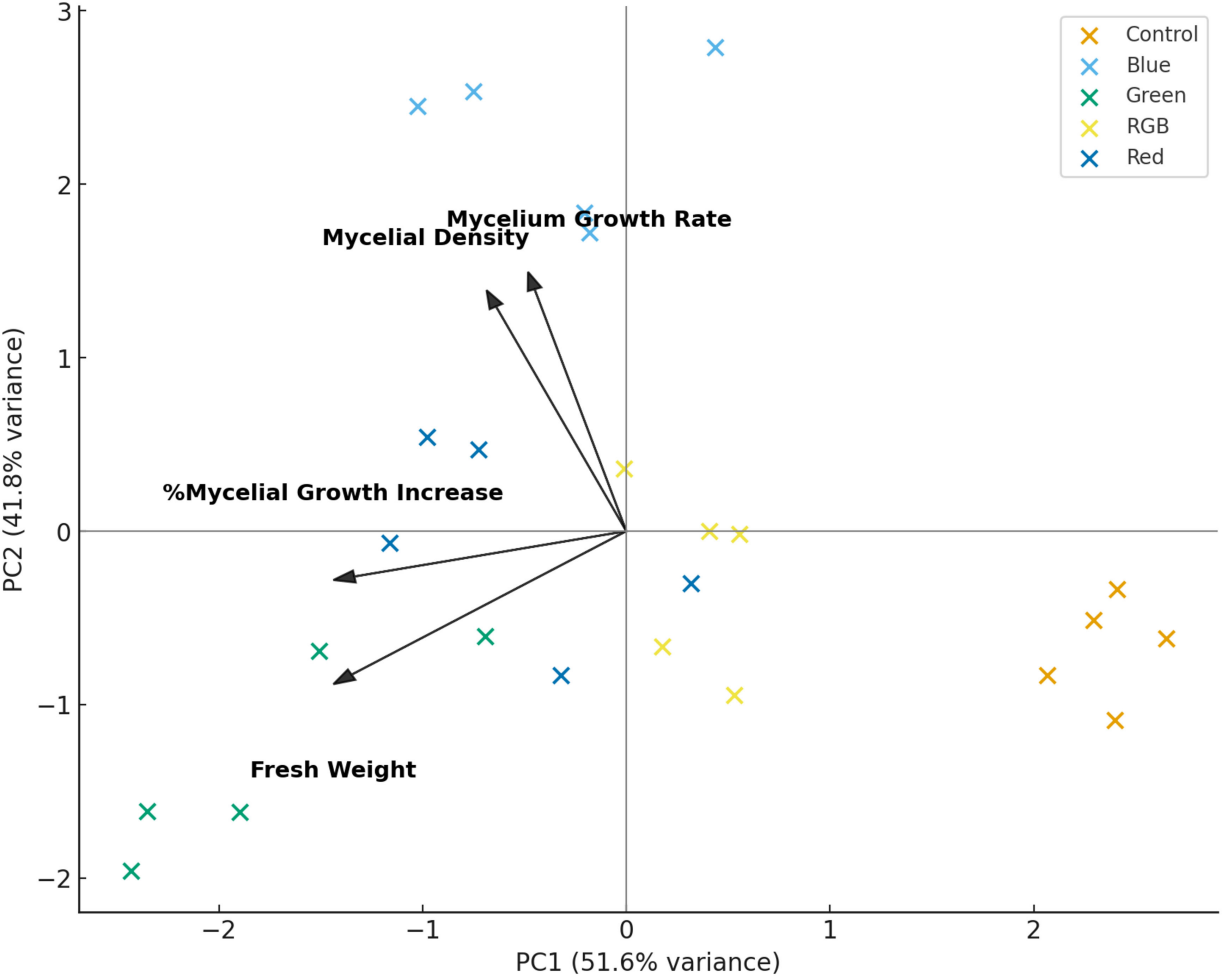
Principal Component Analysis (PCA) biplot of *H. erinaceus* growth parameters under LED light treatments. PC1 (51.6%) and PC2 (41.8%) explain most of the variance. Arrows represent the contribution and direction of each variable (fresh weight, mycelial growth rate, mycelial density, and percentage mycelial growth increase) to the principal components.

The PCA biplot provides an integrative overview of the relationships between fungal growth traits and the effects of different light treatments, as presented in **Figure 8**. The first principal component (PC1) accounted for 51.6% of the total variance, while the second component (PC2) explained an additional 41.8%, together capturing 93.4% of the dataset variation. This high cumulative variance indicates that the selected variables, mycelial growth rate, fresh weight, %mycelial growth increase, and mycelial density, are strongly interrelated and effectively summarized within the two-dimensional PCA space. The vectors show that mycelial growth rate and mycelial density are positively aligned with PC2, while fresh weight and %mycelial growth increase load more strongly along the negative side of PC1. This suggests that PC1 represents a general axis contrasting biomass accumulation with structural development, whereas PC2 distinguishes variation in growth rate and density. Among the treatments, blue and red light cluster in the positive PC2 region, indicating their close association with enhanced growth rate and density. In contrast, green light aligns with negative PC1 values, reflecting its strong association with increased fresh weight and overall growth increment. RGB light is positioned near the origin, indicating a balanced but less pronounced effect across all traits, while the control group clusters toward the positive PC1 axis, signifying lower overall mycelial performance in the absence of light. Collectively, these patterns confirm that light quality significantly modulates fungal growth responses, with red and blue promoting structural development, green favoring biomass accumulation, and RGB producing intermediate outcomes across measured parameters.

### 3.5 Cytotoxic activity against gastrointestinal cancer cell line by SRB assay

The cytotoxic effects of *H. erinaceus* mycelium extracts, cultivated on red sorghum under different light spectra, were assessed against SW480 (colorectal) and HepG2 (liver) cancer cell lines, compared to normal colon cells (CCD-841 CoN) using IC_50_ values as a measure of efficacy. In general, the results demonstrated that mycelial exposure to specific light wavelengths enhanced the anticancer activity of the extracts when compared to both the non-illuminated control and the red sorghum substrate alone. For the SW480 colon cancer cell line, the blue light-treated extract exhibited the strongest cytotoxicity with the lowest IC_50_ value of 133.71 µg/mL, followed closely by RGB (143.38 µg/mL), green light (139.64 µg/mL), and red light (147.47 µg/mL) treatments. Extracts control treatment (159.81 µg/mL) showed comparatively weaker effects. The red sorghum extract alone (without mycelium) showed negligible cytotoxicity (IC_50_ > 200 µg/mL), confirming that the mycelium, rather than the substrate, contributed to the observed effects. Similarly, the HepG2 liver cancer cell line exhibited a comparable pattern of effects to that observed in SW480 cells. According to the blue light treatment showed the most potent effect, yielding an IC_50_ of 114.84 µg/mL, followed by red (126.40 µg/mL). The green and RGB light treatments presented moderate cytotoxicity with IC_50_ values of 140.83 µg/mL and 169.53 µg/mL, respectively. Red sorghum extracts alone exhibited no significant cytotoxic activity (IC_50_ > 200 µg/mL) (**Table 1**). In contrast to the pronounced cytotoxic effects of *H. erinaceus* mycelium observed in the SW480 and HepG2 cancer cell lines, all treatments exhibited relatively low cytotoxicity toward the CCD−841 CoN normal colon epithelial cells, with IC_50_ values consistently above 278.08 µg/mL. This experiment examined the effects of different light treatments (Control, Blue, Green, RGB, and Red) on the concentration (µg/mL) of normal human colon epithelial cells (CCD-841CoN) compared with two cancer cell lines, SW480 and HepG2. Across all treatments, CCD-841CoN exhibited consistently higher concentrations than either SW480 or HepG2, with statistical analysis indicating significant differences in every condition. These results suggest that while light treatments influence both normal and cancer cell lines, CCD-841CoN demonstrates greater viability or resistance than the cancer cell lines under all tested light conditions (**Supplementary Figure S3**). This differential response suggests that the *H. erinaceus* mycelium extracts selectively target cancerous cells while sparing normal ones-an essential characteristic of promising anticancer agents.

**Table 1:** IC_50_ values of *H. erinaceus* mycelium extracts cultivated under different light treatments against SW480, HepG2, and CCD−841 CoN cell lines.

Treatment	SW480 (µg/mL)	HepG2 (µg/mL)	CCD−841 CoN (µg/mL)
Red sorghum	>300	>300	>300
Control	159.81 ± 5.59c	194.80 ± 8.84c	290.76 ± 4.98
Blue	133.71 ± 8.75a	114.84 ± 3.41a	278.08 ± 5.18
Green	145.62 ± 7.21b	140.83 ± 4.38b	289.55 ± 4.28
RGB	143.38 ± 2.56b	169.53 ± 8.72b	283.41 ± 5.11
Red	147.47 ± 5.51b	126.40 ± 3.22ab	280.87 ± 6.03
F-test	**	**	ns
C.V.%	5.18	6.21	4.35

Values are means with standard deviations (*n* = 3). Means with different letters in the same column are significantly different by Duncan’s multiple range tests (*p* < 0.05).

**There were significant differences at *p* < 0.01, and ns = not significant.

**Figure 9:**
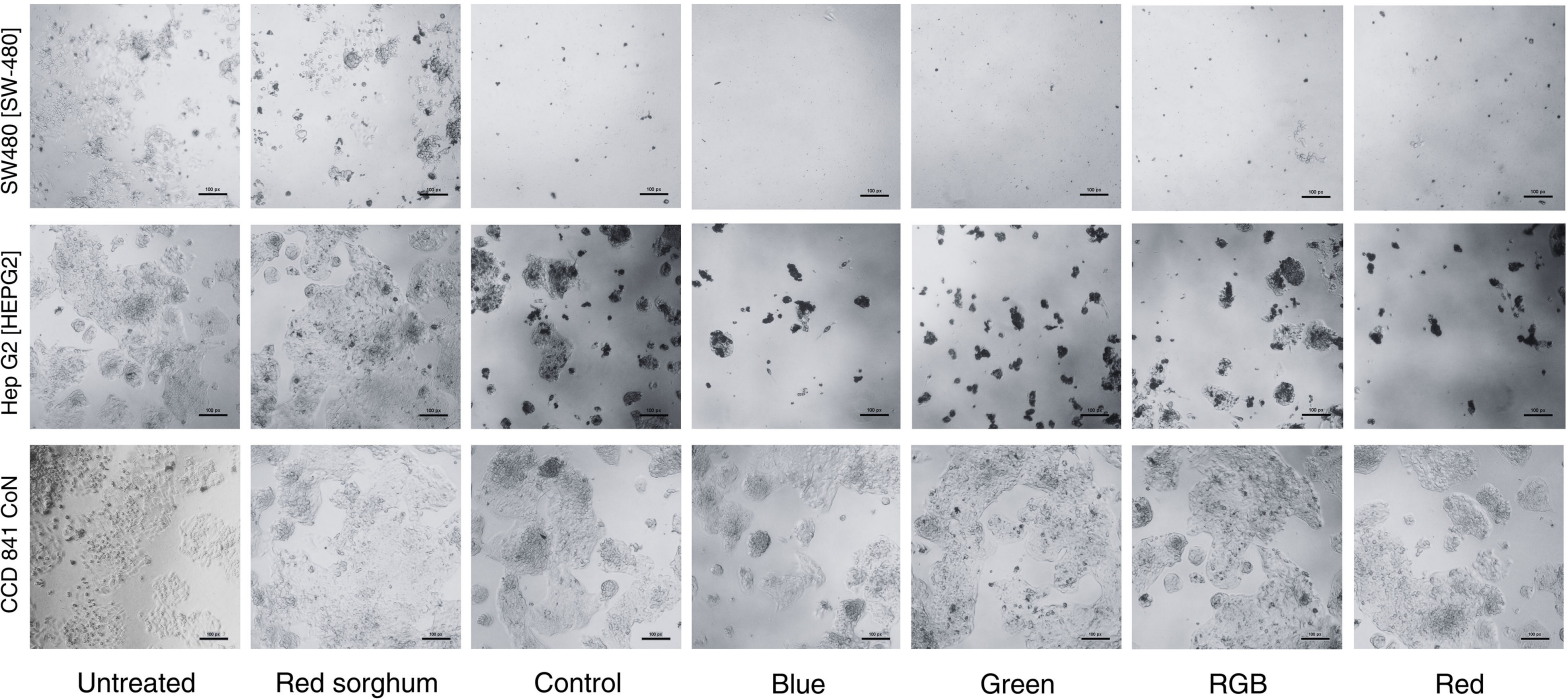
Morphological characteristics in SW480, HepG2, and CCD−841 CoN cell lines following treatment with *H. erinaceus* mycelium cultivated under different light conditions and extracted with ethanol at a concentration of 200 µg/mL, compared to untreated cells.

A study of the cytotoxicity of *H. erinaceus* mycelium against gastrointestinal cancer cells was conducted using extracts cultivated under different light conditions on red sorghum. **Figure 9** presents representative morphological characteristics and cell densities of SW480, HepG2, and CCD−841 CoN cell lines following treatment. Microscopic examination of untreated SW480 cells and those exposed only to red sorghum extract revealed normal epithelial morphology, characterized by dense monolayer growth and strong cell-to-cell adhesion, indicating active and healthy proliferation. However, treatment with 200 µg/mL of *H. erinaceus* mycelium extracts grown under various light conditions (blue, green, red, RGB) resulted in notable morphological alterations and a marked reduction in cell density. The treated SW480 cells appeared shrunken, less adherent, and dispersed, indicating a loss of cellular integrity and disruption of cell–cell interactions. These effects were particularly pronounced in the blue and green light treatments, where viable cells dramatically decreased, leaving only cell debris. The control treatment (mycelium grown without light stimulation) also reduced cell numbers, but to a lesser extent. Among all conditions, blue light-stimulated extracts exhibited the strongest cytotoxic effects. Similarly, HepG2 liver cancer cells displayed epithelial morphology and dense monolayers in the untreated and red sorghum extract groups, indicating no inherent toxicity of the substrate. In contrast, treatment with 200 µg/mL of *H. erinaceus* extracts led to varying degrees of growth inhibition and morphological disruption depending on the light condition. The blue, green, and red light–treated extracts caused the most substantial reduction in cell number and visible signs of shrinkage and structural loss. RGB and control treatments also caused moderate effects, albeit less severe. These observations suggest that light quality during mycelial cultivation significantly influences the biological activity of the extracts, likely by altering metabolite biosynthesis. In contrast to the cancer cell lines, CCD−841 CoN normal colon epithelial cells maintained healthy epithelial morphology under untreated and red sorghum extract conditions, with dense monolayers and intact cell junctions. Treatment with 200 µg/mL of *H. erinaceus* mycelium extracts induced only mild morphological changes in normal cells, with no visible signs of apoptosis, shrinkage, or detachment. Unlike the pronounced cytotoxic effects observed in SW480 and HepG2 cells, the normal cells remained largely unaffected across all treatments. These findings highlight the selective cytotoxicity of *H. erinaceus* extracts, particularly those derived from blue and red light cultivation-which effectively targeted cancer cells while sparing normal ones, thereby suggesting a favorable therapeutic index.

## 4 Discussion

### 4.1 Comparative study of *H. erinaceus* mycelial growth under different light spectra

Light acts as a crucial physical factor in regulating the growth and development of fungal mycelium through the photomorphogenesis process by which light shapes fungal form and structure. In mushrooms, light detection plays a crucial role in various physiological functions, including directing mycelial growth, which is mediated by specific photoreceptors (Yu and Fischer, 2019). As previously reported, mushrooms in the phylum Basidiomycota, such as *H. erinaceus*, utilize various photoreceptors to regulate growth, development, and physiological responses to light. The White-Collar Complex (WCC) detects blue light and plays a key role in gene regulation, while cryptochromes also respond to blue light and influence circadian rhythms and developmental processes (Idnurm et al., 2010; Galindo et al., 2022). Opsins function as light-sensitive proteins that detect green light, affecting spore dispersal and phototaxis. Additionally, phytochromes, which are sensitive to red and far-red light, help fungi adapt their growth to environmental light conditions. These photoreceptors collectively enable Basidiomycota mushrooms to optimize their development in response to light stimuli (Blumenstein et al., 2005; Corrochano, 2019).

This study investigated the effect of light spectra on *H. erinaceus* mycelial growth and biomass. Blue light was most effective, producing the fastest surface coverage, highest fresh weight, and greatest density, while darkness resulted in the slowest and least productive growth. Red, RGB, and green light supported moderate development, though still superior to the control. These findings suggest that blue light significantly enhances biomass production and accelerates growth in the examined fungal species. This aligns with the known function of blue light photoreceptors, which play a key role in regulating fungal development, morphogenesis, and metabolic processes (Idnurm and Heitman, 2005; Corrochano, 2007). Studies indicate that blue light influences fungal growth by activating photoreceptors such as white-collar proteins, which regulate gene expression related to metabolism and cellular differentiation (Yu and Fischer, 2019). The promotion of fast and dense mycelial growth under blue light is attributed to the activation of specific photoreceptors like WC-1. These receptors initiate a cascade of genetic and physiological responses that enhance fungal development (Chiang et al., 2017). These results suggest that exposure to blue light may enhance *H. erinaceus* mycelial biomass accumulation more effectively compared to other light treatments or the absence of light manipulation (control). This observation is consistent with previous findings indicating that blue light plays a crucial role in stimulating fungal growth, morphogenesis, and secondary metabolite production. Several studies have demonstrated that blue light can activate photoreceptor systems in fungi, thereby influencing developmental pathways and metabolic activity (Purschwitz et al., 2009; Fuller et al., 2015). This observation is in accordance with the findings of Zapata et al. (2009), which demonstrated that *Ganoderma lucidum* cultivated under different LED spectra exhibited the highest mycelial biomass under blue light, with white light yielding intermediate results and darkness or red and yellow light producing significantly lower biomass, thereby establishing a clear wavelength dependence for biomass yield in submerged fermentation. Similarly, Jang et al. (2013) showed that LED light quality has a pronounced effect on *Hypsizygus marmoreus* mycelial growth and morphogenesis; across species tested, blue LED light consistently promoted rapid radial growth and dense, compact colonies, often accompanied by enhanced hyphal branching and altered colony texture, while yellow light was least effective. These results demonstrate that blue light is the most favorable spectrum for stimulating mycelial growth and morphological differentiation, highlighting the regulatory role of wavelength-specific photobiological responses in fungi. Supporting this, Park and Jang (2020) demonstrated in *L. edodes* that blue light not only modified fruiting body morphology, producing larger pilei, thicker stipes, and shorter stipe length compared with controls, but also reprogrammed the proteome, with 22 proteins upregulated and 16 downregulated. Notably, enzymes associated with glycolysis (phosphopyruvate hydratase) and one-carbon metabolism (homocysteine S-methyltransferase) were induced. At the same time, proteins involved in the pentose phosphate pathway, proteasomal turnover, and chaperonin function were repressed. These coordinated proteomic shifts suggest that blue light enhances energy metabolism and biosynthetic processes while modulating protein homeostasis to drive fruiting body differentiation. qRT-PCR validation further confirmed these results, providing strong mechanistic evidence that blue light reprograms both metabolic and regulatory pathways to support mushroom morphogenesis.

In contrast, red light appears to have a moderate impact, likely due to its influence on secondary metabolism and enzymatic activity (Purschwitz et al., 2006). This is consistent with the report by Jang et al. (2013), which demonstrated that red light provided moderate stimulation of fruit body formation in *H. marmoreus*. Previous research has shown that red-light exposure can modulate the production of secondary metabolites in fungi, affecting their overall growth and bioactivity (Fuller et al., 2015). Similarly, Chiang et al. (2017) demonstrated that red light induced the highest levels of biomass and mannitol in *C. militaris*. A green light generally has a weaker effect on fungal growth, as many organisms possess fewer photoreceptors sensitive to green wavelengths (Tisch and Schmoll, 2010). However, certain fungal species may still exhibit minor physiological responses to green light, depending on their environmental adaptations (Dias et al., 2020). The intermediate results observed under RGB lighting indicate that while a mixture of wavelengths can support growth, it may not be as effective as exposure to a single dominant wavelength, like blue light (Yu et al., 2023). Research on fungal photobiology suggests that combining different wavelengths could lead to competing or overlapping signaling effects, influencing growth and metabolite production in complex ways (Lim et al., 2024). The relatively low fresh weight and growth increase in the control condition highlight the limited capacity for mycelial development under ambient, uncontrolled light conditions. These findings emphasize the importance of light spectrum selection in optimizing fungal cultivation systems. They also align with the broader understanding that light functions not only as an environmental cue but also as a developmental regulator in fungi, influencing cellular processes essential for biomass accumulation (Purschwitz et al., 2006). Notably, light quality markedly affected the mycelial density of *H. erinaceus*. Among the evaluated treatments, blue light produced the greatest mycelial density, significantly higher than that of the dark control. This finding suggests that short-wavelength illumination strongly promotes biomass accumulation and hyphal compactness, presumably through the activation of blue-light photoreceptors, which govern photomorphogenesis, enzyme secretion, and secondary metabolism in basidiomycetes (Kamada et al., 2010). RGB and red light yielded intermediate responses, while green light exerted a minor stimulatory effect. In this study, the ImageJ software was employed to evaluate mycelial density, as ImageJ-based mycelial density analysis is a quantitative image analysis technique that measures the optical density of mycelia from digital images. The analysis is performed by calculating the percentage of area coverage and pixel intensity values, which effectively indicate the physical extent of mycelial growth (Lee et al., 2021). Although the ImageJ software allows quantitative assessment of visible mycelial coverage and surface area, it primarily measures morphological features rather than physiological dynamics. Therefore, the observed increases in %Area and calculated density may not fully reflect intracellular metabolic activity, differentiation potential, or the initiation of fruiting primordia formation. Because fungal growth involves multidirectional expansion and complex physiological regulation, such as alterations in oxidative metabolism, these findings suggest that blue light not only enhances the visible compactness of *H. erinaceus* mycelia but may also trigger underlying metabolic pathways that sustain active growth. Acknowledging the inherent limitations of image-based quantification thus deepens the interpretation of these results and emphasizes the necessity of integrating complementary physiological or molecular approaches when investigating fungal responses to environmental light cues (Casas-Flores et al., 2006).

Light quality emerged as a decisive factor shaping mycelial development, as evidenced by the strong positive correlations among growth parameters (*r* = 0.97–0.99), particularly between growth rate and density, suggesting that accelerated hyphal extension is inherently coupled with structural compactness. The PCA analysis, which explained 93.4% of the total variance, further revealed distinct clustering of treatments: blue- and red-light groups aligned closely with vectors for growth rate and density, indicating that these spectral regions promote both rapid expansion and dense mycelial networks. In contrast, green light was positioned in association with fresh weight, reflecting a divergent growth strategy favoring biomass accumulation over compact architecture, while the RGB treatment clustered nearer to the control, underscoring its limited efficacy in modulating fungal physiology. Hierarchical clustering corroborated these findings, placing blue and red in a distinct cluster apart from green, RGB, and the control, thereby emphasizing their convergent stimulatory effects. Mechanistically, the observed enhancement under blue and red illumination may be attributed to activation of photoreceptor-mediated signaling pathways such as the WCC for blue light and phytochrome-like receptors for red light that regulate fungal morphogenesis and secondary metabolism. Conversely, the weaker yet specific effect of green light suggests the involvement of less-characterized photoreceptive mechanisms that may divert resources toward biomass rather than structural density. Collectively, these results highlight that spectral quality does not simply alter growth magnitude but reprograms developmental trajectories, reinforcing earlier reports on the role of light in fungal morphogenesis and physiology (Miles and Chang, 2004).

### 4.2 Anticancer activity against gastrointestinal cancer cell lines

The cytotoxic evaluation of *H. erinaceus* mycelium extracts cultivated on red sorghum under varying light spectra revealed selective anticancer activity against SW480 colorectal and HepG2 liver cancer cells, while sparing normal colon epithelial cells (CCD-841 CoN). Blue light treatment yielded the most potent effects, followed by green, RGB, and red light, all outperforming non-illuminated controls and the red sorghum substrate alone. Importantly, all treatments exhibited markedly lower cytotoxicity toward normal cells. These findings strongly suggest that light quality during cultivation significantly influences the anticancer potential of *H. erinaceus* mycelium, likely by enhancing the biosynthesis of bioactive metabolites such as phenolics, polysaccharides, and possibly erinacines, which are known for their apoptotic and antiproliferative effects (Bhosale et al., 2020). The notably low IC_50_ values observed in both cell lines following blue light exposure suggest that this wavelength most effectively stimulates the production of cytotoxic compounds. Green and red light also contributed to enhanced bioactivity, though to a slightly lesser extent (Wang et al., 2020). The RGB treatment showed intermediate effects in SW480 cells and was relatively less effective in HepG2, indicating that the light spectrum may influence metabolite profiles differently depending on the fungal developmental stage or targeted cell type. The bioactive compounds produced from mycelium are presumed to exert anticancer effects through mechanisms similar to those reported for vanadium complexes (Ghosh and Ghosh, 2021). These mechanisms include direct DNA damage through binding to DNA strands, leading to cell cycle arrest and apoptosis; induction of oxidative stress via increased reactive oxygen species (ROS), resulting in damage to proteins and nucleic acids; activation of the mitochondrial apoptotic pathway involving Cytochrome c (cyc. c) release and caspase activation; and cell cycle arrest at the G_2_/M phase, which inhibits cancer cell proliferation. Therefore, the significantly reduced IC_50_ values observed in mycelial extracts cultivated under blue light may reflect an enhanced biosynthesis of metabolites acting through these specific anticancer pathways (Kowalski et al., 2020). A study by Dong et al. (2012), which investigated the effects of light wavelengths on cordycepin production in *C. militaris* under different illumination conditions, reported that exposure to blue light significantly increased cordycepin levels compared to non-illuminated controls. This effect is likely attributable to blue light-mediated metabolic rerouting that favors the biosynthesis of cordycepin precursors, the activation of specific enzymes, and the upregulation of genes associated with cordycepin biosynthesis. Notably, transcriptomic profiling conducted on *P. ostreatus* and found that blue light exposure significantly upregulated genes associated with glycolysis and the pentose phosphate pathway, both of which are linked to secondary metabolite biosynthesis. In contrast, red light exhibited a downregulating effect on these pathways (Xie et al., 2018). These findings suggest that blue light can stimulate key metabolic processes, leading to enhanced production of bioactive compounds, such as cytotoxic metabolites, thereby supporting the observed increase in anticancer activity under blue light conditions. Although the present study demonstrated notable cytotoxic activity, its anticancer potency was comparatively lower than that reported for other medicinal mushrooms, particularly *G. lucidum*. Toson et al. (2025) previously reported that the fruiting bodies of *G. lucidum* exerted pronounced inhibitory effects against hepatocellular carcinoma (HepG2) cell lines, with IC_50_ values of 72.32 µg/mL when using a concentration of 100 µg/mL, the cell viability of the hepatic tumor cell line was 40%. While fruiting body extracts can yield stronger anticancer activity, their large-scale application is often constrained by the considerably longer cultivation period required compared with mycelial cultures (Zhou et al., 2012).

In the study of morphological characteristics in SW480, HepG2, and CCD-841 CoN cell lines, *H. erinaceus* mycelium extracts cultivated on red sorghum under different light spectra exhibited selective cytotoxic effects against gastrointestinal cancer cells. The results from this study reinforce the growing body of evidence supporting the anticancer potential of *H. erinaceus*, particularly its mycelial extracts cultivated under controlled environmental conditions. The significant reduction in cell viability and the morphological disruptions observed in SW480 and HepG2 cancer cell lines, characterized by loss of monolayer integrity, cell shrinkage, and fragmentation, suggest a direct cytotoxic impact that interferes with fundamental processes such as cell adhesion and survival. These cytological features are indicative of apoptotic cell death, a known outcome of bioactive compound action from fungi, including *H. erinaceus*. Previous studies have identified polysaccharides, phenolic compounds, and erinacines as major constituents of *H. erinaceus* with anticancer properties. These compounds have been shown to trigger oxidative stress, cell cycle arrest, and caspase-dependent apoptosis in cancer cells (Kah Hui et al., 2008; Friedman, 2015). The increased cytotoxicity observed in extracts from blue light-stimulated mycelium supports the hypothesis that light quality modulates fungal secondary metabolism. These findings are consistent with the study of Wang et al. (2020), which reported that light quality was shown to enhance the levels of carbohydrate-active enzymes and oxidative enzymes, thereby further supporting the notion that photoregulation can be harnessed to effectively control metabolite biosynthesis in *P. ostreatus*. Blue light, in particular, has been shown to activate photoreceptors that regulate genes involved in the shikimate and phenylpropanoid pathways, enhancing the biosynthesis of phenolics, flavonoids, and anthocyanin compounds, well known for their antioxidant and anti-proliferative properties (Xie et al., 2018; Park, 2022). Moreover, recent transcriptomic studies on *P. ostreatus* and other edible fungi have demonstrated that blue and red-light exposure significantly upregulates genes linked to carbohydrate-active enzymes (CAZymes), oxidative enzymes, and secondary metabolism, which are responsible for producing compounds that may interfere with cancer cell signaling pathways (Wang et al., 2020; Park, 2022). Thus, your findings align with this mechanistic understanding, indicating that blue light cultivation enhances the bioefficacy of fungal extracts by enriching the pool of therapeutically relevant metabolites.

Microscopic observations reinforced the quantitative results, showing that untreated SW480 and HepG2 cells, as well as those exposed only to sorghum extract, preserved normal epithelial morphology with intact monolayers. In contrast, exposure to *H. erinaceus* mycelial extracts, particularly those derived from blue and green light cultivation, caused marked shrinkage, loss of adhesion, and cellular fragmentation, morphological hallmarks of apoptosis that reflect disruption of cell survival pathways. Normal colon epithelial cells (CCD-841 CoN), however, retained healthy morphology under all treatments, exhibiting only minor alterations even at higher extract concentrations. Taken together with the growth analyses, these findings indicate that light spectrum exerts a dual influence: it not only shapes fundamental mycelial development but also enhances the bioactivity of derived extracts against cancer cells. From an applied standpoint, blue light represents the most effective spectrum, providing superior growth performance and the strongest cytotoxic activity, while red and green light can be employed to adjust metabolite profiles according to therapeutic objectives. Overall, the results highlight the value of wavelength-specific LED cultivation strategies to maximize both yield and medicinal potential in *H. erinaceus*.

## 5 Conclusion

This study suggests that the light spectrum, particularly blue light, can influence the growth and potential bioactivity of *H. erinaceus* mycelium cultivated on a red sorghum substrate. Blue light was associated with increased mycelial biomass, density, and faster colonization, as well as enhanced cytotoxic effects against selected gastrointestinal cancer cell lines. Red and green lights also showed moderate improvements compared to the control. These outcomes suggest that light quality may play a role in modulating fungal development and metabolite production. However, the study has several limitations. The findings are based on *in vitro* assays and specific culture conditions, which may not fully reflect complex environmental or clinical settings. Further studies are needed to confirm these effects *in vivo*, identify the active compounds, and explore the regulatory pathways involved. Despite these limitations, the results provide a useful starting point for optimizing cultivation conditions to enhance the functional properties of *H. erinaceus*.

## Data Availability

The original contributions presented in the study are included in the article/**Supplementary Material**. Further inquiries can be directed to the corresponding author.
