# Supplementary material for: LED light spectra influence the stimulation of mycelial growth and anticancer activity in Hericium erinaceus mycelium
Source: Front Fungal Biol. 2025 Nov 26;6:1684852. doi: 10.3389/ffunb.2025.1684852 (PMC12689372; doi:10.3389/ffunb.2025.1684852)
Supplement: Supplementary file 1 [file DataSheet1.pdf]

Supplementary Material

1 Supplementary Data

The three cell lines used in this study were obtained from the ATCC (American Type Culture Collection) and are described as follows:

1. SW480 [CCL-228] cells isolated from the large intestine of a Dukes C colorectal cancer patient can be used in cancer research.

Organism: *Homo sapiens*, human

Tissue: Large intestine; Colon

Age: 50 years

Gender: Male

Morphology: epithelial

Growth properties: Adherent

Disease: Adenocarcinoma; Colorectal; Dukes' type B

2. Hep G2 [HB-8065] is a cell line exhibiting epithelial-like morphology that was isolated from a hepatocellular carcinoma of a 15-year-old, White, male youth with liver cancer. The cell line was deposited by the Wistar Institute and is a suitable transfection host. Expression markers include insulin; insulin-like growth factor II (IGF II).

Organism: *Homo sapiens*, human

Tissue: Liver

Age: 15 years

Gender: Male

Morphology: epithelial-like

Growth properties: Adherent

Disease: Carcinoma; Hepatocellular

3. CCD 841 CoN [CRL-1790] is an adherent cell line isolated from the colon tissue of a healthy donor. This line resembles epithelial cells; however, the cells do not contain keratin or definitive evidence of epithelial origin. These cells are non-malignant and can be used as a normal gastrointestinal (GI) functional cell model to investigate inflammatory and cytokine secretion mechanisms.

Organism: *Homo sapiens*, human

Cell Type: colonocyte

Tissue: Large intestine; Colon

Age: 21 weeks of gestation

Gender: Female

Morphology: epithelial

Growth properties: Adherent

Disease: Normal

2 Supplementary Figures and Tables

2.1 Supplementary Figures

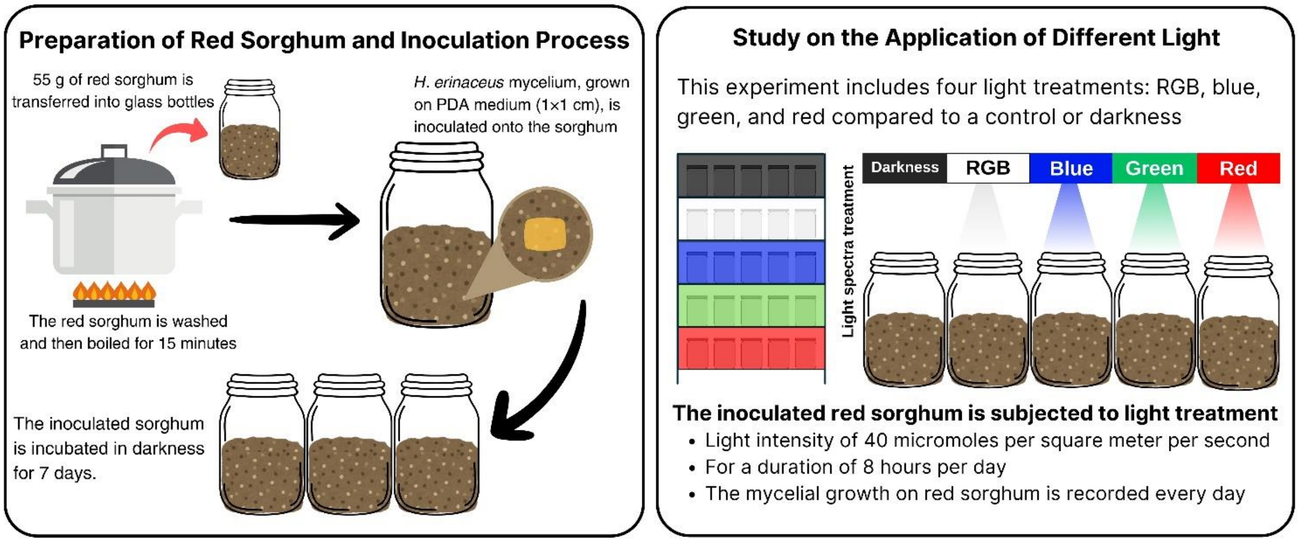

Supplementary Figure 1. This figure illustrates the step-by-step process of preparing red sorghum as a solid substrate for *H. erinaceus* mycelium cultivation. After washing and boiling, the red sorghum is inoculated with mycelial plugs grown on PDA medium and pre-incubated in darkness for 7 days. Following this period, the inoculated substrates are exposed to four different light treatments, RGB, blue, green, and red, compared to a control (darkness)

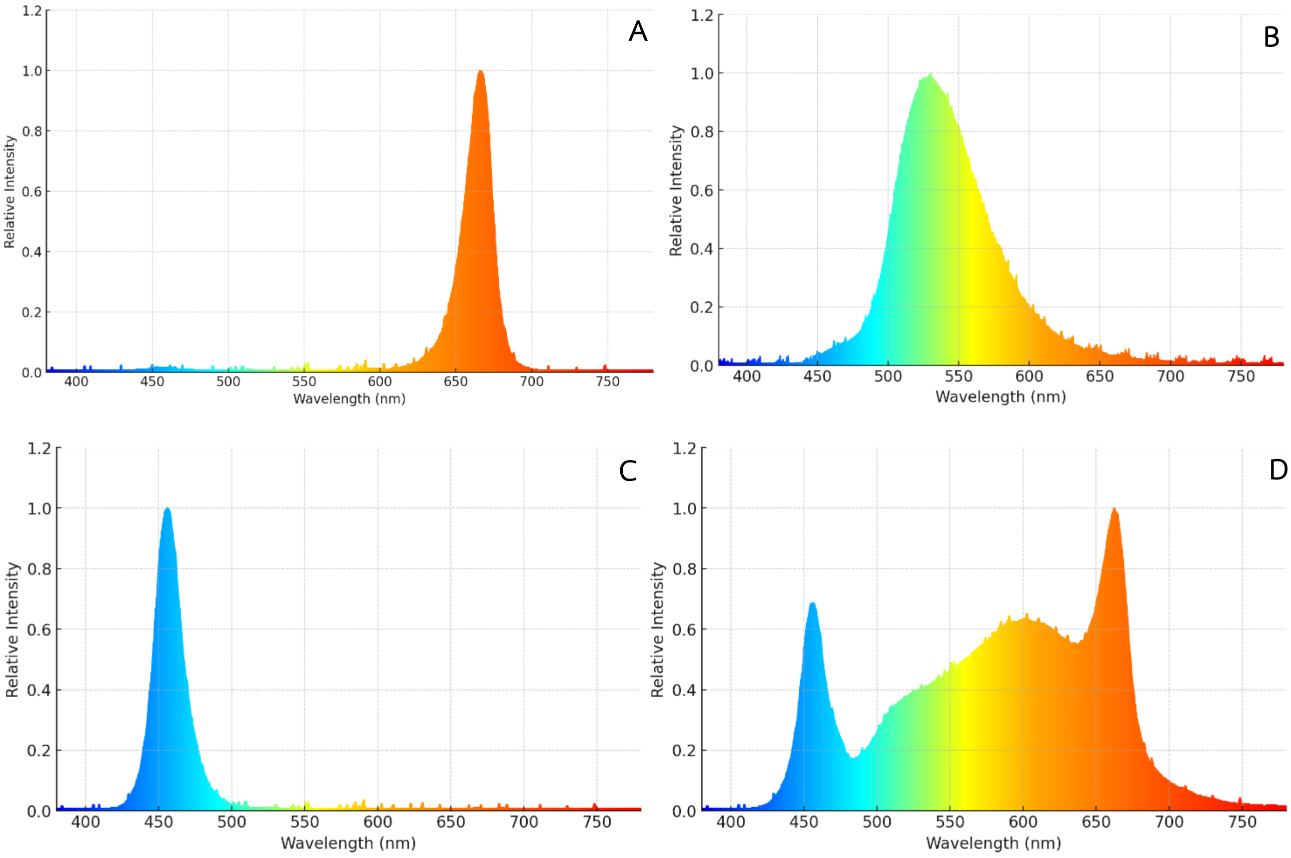

Supplementary Figure 2. Light spectrum graphs: A) Red light spectrum, B) Green light spectrum, C) Blue light spectrum, and D) RGB light spectrum. The spectral intensity of each light source was verified using a quantum light meter to ensure consistency

Supplementary Table 1. Mycelium growth parameters under different light treatments

Treatment	Growth rate (day)	Mycelium weight (g)	Mycelial growth increase (%)	Mycelial Density (g/cm^2)
Control	30±0.14d	3.83±0.18d	6.96±0.32d	0.195±0.021e
Blue	15±0.48a	6.75±0.65a	12.28±1.18a	0.344±0.072a
Green	20±0.14c	4.86±0.39c	8.83±0.70c	0.247±0.056d
Red	18±0.58b	5.60±0.33b	10.18±0.60b	0.285±0.094b
RGB	18±0.55b	5.06±0.18c	9.19±0.32c	0.257±0.074c
F-test	**	**	**	**
C.V.%	4.89	6.31	6.31	10.66

Values are means with standard deviations (n = 5). Means with different letters in the same column are significantly different by Duncan's multiple range tests ($p < 0.05$).
** There were significant differences at $p < 0.01$

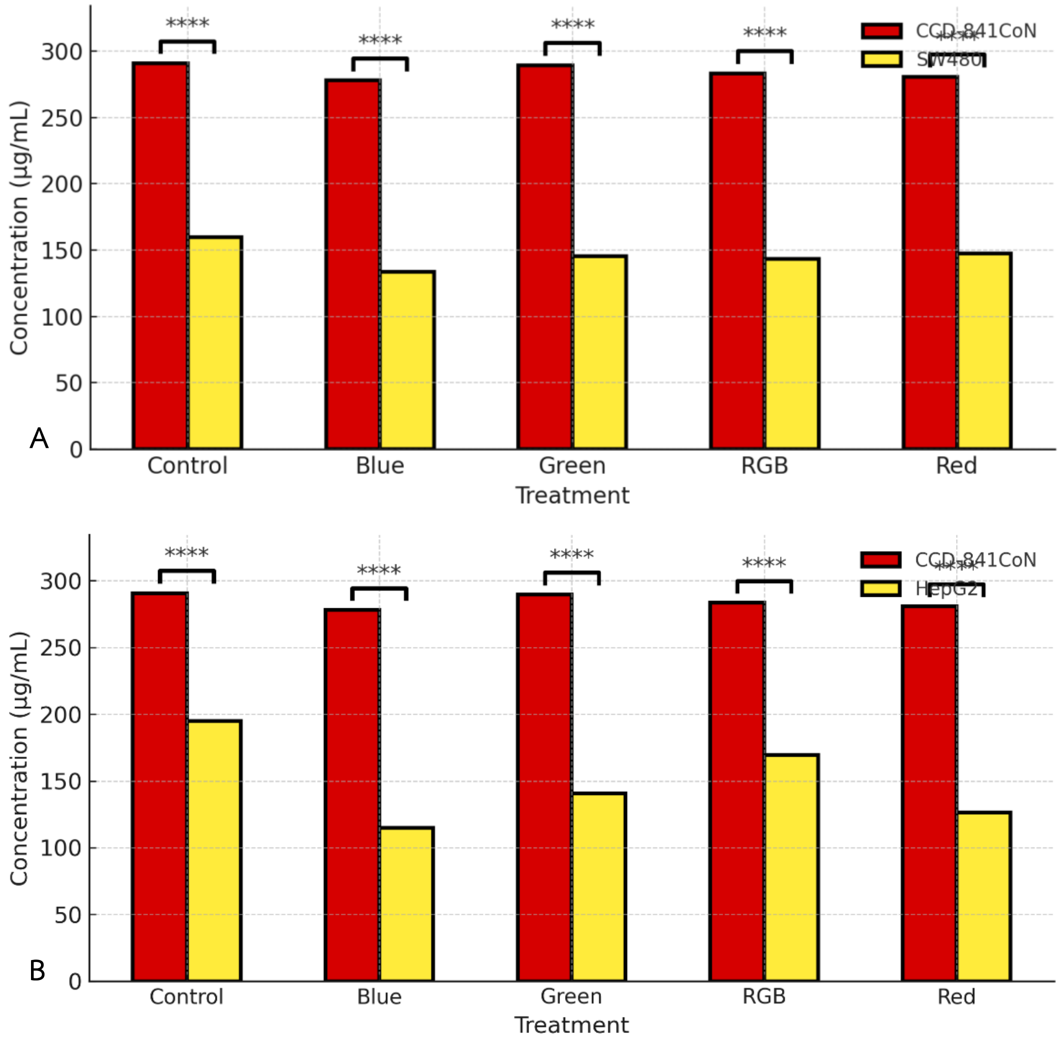

Supplementary Figure 3. Comparative effects of CCD-841CoN on the viability of A) SW480 and B) HepG2 cells under different light treatments
